# Alterations of alveolar type II cells and intraalveolar surfactant after bronchoalveolar lavage and perfluorocarbon ventilation. An electron microscopical and stereological study in the rat lung

**DOI:** 10.1186/1465-9921-8-40

**Published:** 2007-06-05

**Authors:** Mario Rüdiger, Sebastian Wendt, Lars Köthe, Wolfram Burkhardt, Roland R Wauer, Matthias Ochs

**Affiliations:** 1 Clinic for Neonatology, Charité Universitätsmedizin Berlin, Campus Mitte, Berlin, Germany; 2 Clinic for Pediatrics, Pädiatrie IV – Neonatologie; Medical University of Innsbruck, Innsbruck, Austria; 3 Department of Anatomy, Division of Electron Microscopy, Georg-August-University, Göttingen, Germany; 4 Institute of Anatomy, Experimental Morphology, University of Bern, Bern, Switzerland

## Abstract

**Background:**

Repeated bronchoalveolar lavage (BAL) has been used in animals to induce surfactant depletion and to study therapeutical interventions of subsequent respiratory insufficiency. Intratracheal administration of surface active agents such as perfluorocarbons (PFC) can prevent the alveolar collapse in surfactant depleted lungs. However, it is not known how BAL or subsequent PFC administration affect the intracellular and intraalveolar surfactant pool.

**Methods:**

Male wistar rats were surfactant depleted by BAL and treated for 1 hour by conventional mechanical ventilation (*Lavaged-Gas*, n = 5) or partial liquid ventilation with PF 5080 (*Lavaged-PF5080*, n = 5). For control, 10 healthy animals with gas (*Healthy-Gas*, n = 5) or PF5080 filled lungs (*Healthy-PF5080*, n = 5) were studied. A design-based stereological approach was used for quantification of lung parenchyma and the intracellular and intraalveolar surfactant pool at the light and electron microscopic level.

**Results:**

Compared to *Healthy*-lungs, *Lavaged*-animals had more type II cells with lamellar bodies in the process of secretion and freshly secreted lamellar body-like surfactant forms in the alveoli. The fraction of alveolar epithelial surface area covered with surfactant and total intraalveolar surfactant content were significantly smaller in *Lavaged*-animals. Compared with *Gas*-filled lungs, both *PF5080*-groups had a significantly higher total lung volume, but no other differences.

**Conclusion:**

After BAL-induced alveolar surfactant depletion the amount of intracellularly stored surfactant is about half as high as in healthy animals. In lavaged animals short time liquid ventilation with PF5080 did not alter intra- or extracellular surfactant content or subtype composition.

## Background

The pulmonary surfactant system covers the alveolar surface and prevents end-expiratory alveolar collapse by reducing surface tension. The total surfactant content can be divided into an intraalveolar and an intracellular pool. According to recent models [1], intracellular surfactant is found in specific storage organelles (lamellar bodies) within alveolar type II cells. Intraalveolar surfactant metabolism involves transformation of freshly secreted lamellar body-like forms into tubular myelin with characteristic lattice-like appearance, insertion of surfactant material into the surface layer and conversion of "spent" surfactant into unilamellar vesicles which are recycled or degraded.

Repeated bronchoalveolar lavage (BAL) induces alveolar surfactant depletion [2] and is often used in animal models to induce acute lung injury and to study therapeutic interventions [3]. Despite of the frequent use, little is known about the immediate effects of BAL on the endogenous surfactant system. Differential centrifugation of intraalveolar surfactant material obtained by BAL reveals two subtypes: surface active large aggregates, ultrastructurally mainly corresponding to lamellar body-like forms, multilamellar vesicles and tubular myelin, and inactive small aggregates, ultrastructurally mainly corresponding to unilamellar vesicles [4]. Thus, BAL most likely reduces the intraalveolar surfactant content; the fate of intracellular pool, however, remains speculative.

Surfactant deficiency presents as respiratory distress and often requires mechanical ventilation. Disturbed intraalveolar surface tension can be improved by intratracheal application of surface active agents such as exogenous surfactant [3,5] or perfluorocarbons (PFC) [6]. PFC associated gas exchange improves oxygenation of surfactant depleted animals, however, data regarding the effect of PFC on the surfactant secretion and synthesis are controversial and could depend on the type of PFC that is studied [7-9].

For an appropriate interpretation of physiological data obtained from surfactant depleted animals it should be known how the experimental protocol of BAL and mechanical ventilation [2] alters the pulmonary surfactant. Furthermore, it is of clinical interest to know whether short time contact with intraalveolar PFC modulates the BAL induced response. We therefore investigated the impact of BAL and subsequent short term partial liquid ventilation upon intracellular and intraalveolar surfactant in a rat model. Changes that are caused by lavage and subsequent liquid ventilation were analyzed at the light microscopic as well as at the electron microscopic level, using a previously described design-based stereological approach for quantification of lung parenchymal architecture and the intracellular and intraalveolar surfactant pool [10-12]. The unique feature of this approach is that it allows the analysis of the intraalveolar as well as the intracellular surfactant in its natural microorganization and localization within the lung [13].

## Materials and methods

### Animals

In total, 20 male Wistar rats at an age of 2 months were studied. Care of the animals was in accordance with guidelines for ethical animal research. The study was approved by the local Review Board. The reason for choosing 5 animals per group in a stereological study is that if a parameter is found to change in one direction in all 5 cases, then the probability that this is due to chance is p = (1/2)^5 ^< 0.05, thus making the experiment conclusive [14].

Rats were anaesthetized with Ketamin (10 mg/kg) and Pentobarbital (20 mg/kg) intraperitoneally. A catheter was placed intravenously and a glucose electrolyte mixture (20 ml) containing Fentanyl (20 μg), Pancuronium (0.4 mg) and Midazolam (2 mg) was given at a rate of 2 ml/h. A tube with side port for PFC application was inserted via tracheostomy. All animals were placed on a pressure controlled ventilation (BP 2001, Bear Medical Systems, Inc., Palm Springs, Calif., USA) with the following settings: PIP 10, PEEP 3 cmH_2_O, FiO_2 _0.5, inspiratory time 0.4 sec, frequency 60/min.

### Experimental protocol

To obtain control data for electron microscopic analysis of pulmonary surfactant parameters, 10 healthy animals were randomized into two control groups. The *Healthy-Gas *group (n = 5) received an air bolus of 30 ml/kg via the side port and animals were sacrificed after five minutes of conventional ventilation. PF 5080 (C_8_F_18_, molecular weight 438, density 1.77 g/ml, viscosity 0.75 cSt, surface tension 15 mN/m, vapor pressure 61 torr), a perfluorocarbon that has been previously used in cell and animal studies [15,16], was obtained from 3M Germany (Neuss, Germany). The *Healthy-PF5080 *group (n = 5) received 30 ml/kg of PF 5080 via the side port of the endotracheal tube within 1 minute. To verify a homogenous distribution of PF5080 ventilation was continued for 5 minutes (in both *healthy *groups) and the animals were sacrificed thereafter with an overdose of pentobarbital.

To study the impact of BAL and subsequent partial liquid ventilation (PLV) upon the intra cellular and intraalveolar surfactant, another 10 animals were randomized into two groups: *Lavaged-Gas *(n = 5) and *Lavaged-PF5080 *(n = 5). To monitor arterial blood gases, an arterial line was placed and connected with a pressure transducer for recording of blood pressure. Thereafter, animals were placed into an incubator to keep body temperature constant. ECG was measured continuously using Servo SMV 178 monitor (Hellige, Germany). To induce intraalveolar surfactant depletion, the bronchoalveolar lavage (BAL) protocol of Lachmann et al. [2] was slightly modified. In detail, inspiratory pressure was increased up to 20 cmH_2_O, other ventilatory parameters were kept constant. 30 ml of warmed saline was administered via the endotracheal tube within 30 sec, ventilation was continued for another 30 sec and lavage fluid was withdrawn thereafter. Animals were allowed to recover for 1 minute before the lavage procedure was repeated. After 5 lavage procedures arterial blood gases were obtained, if PaO_2 _was above 100 mmHg the lavage procedure was repeated. When a PaO_2 _lower than 100 mmHg was achieved animals were kept on conventional ventilation and after 15 minutes blood gases were checked to exclude spontaneous improvement of oxygenation. If PaO_2 _had increased above 100 mmHg, lavage procedure was repeated, otherwise the experimental protocol started.

All parameters were obtained at baseline, thereafter treatment according to randomization was initiated. In the *Lavaged-Gas *group conventional mechanical ventilation was continued with the same ventilatory setting. In the *Lavaged-PF5080 *animals partial liquid ventilation was initiated. PF 5080 was administered intratracheally via sideport at a rate of 30 ml/h until a liquid meniscus was visible in the tube at end-expiration. Thereafter, PF5080 was given at about 9 ml/h to compensate for evaporative losses and to verify a continuous PFC-filling of the lung [17]. Animals were sacrificed after 60 minutes of mechanical ventilation with an overdose of pentobarbital.

Samples (150 μl) of arterialized blood were drawn to determine blood gases (ABL 505, Radiometer Med. A/S, Denmark) prior to lavage, at baseline (0 min) and at 5, 10, 20, 30, 60 minutes of treatment.

To measure tidal volume in ventilated animals, the flow sensor (CO_2_SMO; Novametrix, USA) was placed between the T piece of the ventilator and the endotracheal tube. Measurements were performed prior to lavage, at baseline and 30 and 60 minutes of therapy.

### Fixation, sampling and processing

All lungs (n = 5 per group) underwent light and electron microscopical as well as stereological analysis. After sacrificing the animals ventilation was stopped and a continuous positive airway pressure of 5 cmH_2_O was administered to prevent lungs from collapsing during the fixation procedure. The abdominal cavity was opened and animals were exsanguinated. After opening the thoracic cavity, the pulmonary artery was canulated and the lung was perfused with saline containing 1 IE Heparin per ml with a hydrostatic pressure of 15 cmH_2_O up until the lungs were blood free. Thereafter, lung fixation was performed by vascular perfusion with 1.5% glutaraldehyde and 1.5% formaldehyde (prepared from freshly depolymerized paraformaldehyde) in 0.15 M Hepes buffer [18]. At the end of perfusion the main bronchus and the pulmonary vessels were clamped. The organ was stored in cold fixative until further processing was performed [10]. The volume of the lungs (*V*(*lung*)) was determined by fluid displacement [19]. Using a systematic uniform random sampling protocol [20], samples that by definition represent all parts of the organ equally well were taken for light and electron microscopical analysis. Light microscopical samples were osmicated, bloc-stained in uranyl acetate, dehydrated in acetone and embedded in glycol methacrylate (Technovit 7100, Heraeus Kulzer, Wehrheim, Germany). Electron microscopical samples were osmicated, bloc-stained in uranyl acetate, dehydrated in acetone and embedded in Araldite.

### Stereological analysis

Quantification by means of design-based stereology was performed with a computer-assisted stereology toolbox (CAST 2.0, Olympus, Ballerup, Denmark) connected to a Zeiss Axioskop light microscope (Carl Zeiss, Göttingen, Germany) and with an image analysis system (Analysis 3.1, SIS, Münster, Germany) connected to a Leo EM 900 transmission electron microscope (Leo, Oberkochen, Germany) equipped with a digital camera (MegaView III, SIS, Münster, Germany). The following parameters were estimated using established design-based stereological methods [10,11,21]: At the light microscopic level, the volume fraction of parenchyma per lung (*V*_*V*_(*par/lung*)), the volume fraction of septal tissue (*V*_*V*_(*sep/par*)) and airspace lumen per parenchyma (*V*_*V*_(*air/par*)) was estimated by point counting. The alveolar epithelial surface area (*S*(*alvepi*)) was estimated by intersection counting. The mean thickness of the alveolar septum (τ¯
 MathType@MTEF@5@5@+=feaafiart1ev1aaatCvAUfKttLearuWrP9MDH5MBPbIqV92AaeXatLxBI9gBaebbnrfifHhDYfgasaacH8akY=wiFfYdH8Gipec8Eeeu0xXdbba9frFj0=OqFfea0dXdd9vqai=hGuQ8kuc9pgc9s8qqaq=dirpe0xb9q8qiLsFr0=vr0=vr0dc8meaabaqaciaacaGaaeqabaqabeGadaaakeaaiiGacuWFepaDgaqeaaaa@2E90@(*sep*)) was estimated as twice the alveolar septal volume divided by alveolar epithelial surface area. The volume-weighted mean volume of distal air spaces (ν¯V
 MathType@MTEF@5@5@+=feaafiart1ev1aaatCvAUfKttLearuWrP9MDH5MBPbIqV92AaeXatLxBI9gBaebbnrfifHhDYfgasaacH8akY=wiFfYdH8Gipec8Eeeu0xXdbba9frFj0=OqFfea0dXdd9vqai=hGuQ8kuc9pgc9s8qqaq=dirpe0xb9q8qiLsFr0=vr0=vr0dc8meaabaqaciaacaGaaeqabaqabeGadaaakeaaiiGacuWF9oGBgaqeamaaBaaaleaacqWGwbGvaeqaaaaa@2FE4@(*air*)) was estimated by the point-sampled intercepts method. The number of alveolar type II cells per lung (*N*(*typeII/lung*)) was estimated by the physical disector method and the number-weighted mean volume of type II cells (ν¯N
 MathType@MTEF@5@5@+=feaafiart1ev1aaatCvAUfKttLearuWrP9MDH5MBPbIqV92AaeXatLxBI9gBaebbnrfifHhDYfgasaacH8akY=wiFfYdH8Gipec8Eeeu0xXdbba9frFj0=OqFfea0dXdd9vqai=hGuQ8kuc9pgc9s8qqaq=dirpe0xb9q8qiLsFr0=vr0=vr0dc8meaabaqaciaacaGaaeqabaqabeGadaaakeaaiiGacuWF9oGBgaqeamaaBaaaleaacqWGobGtaeqaaaaa@2FD4@(*typeII*)) was estimated by the planar rotator method. At the electron microscopic level, the surface fraction of alveolar epithelium covered with surfactant (*S*_*S*_(*surf/alvepi*)) was estimated by intersection counting. The volume fractions of intraalveolar surfactant subtypes, namely lamellar body-like forms (*V*_*V*_(*lbl/surf*)), tubular myelin (*V*_*V*_(*tm/surf*)), multilamellar vesicles (*V*_*V*_(*mv/surf*)) and unilamellar vesicles (*V*_*V*_(*uv/surf*)), were estimated by point counting. For evaluation of the intracellular surfactant pool, the volume fraction of lamellar bodies per type II cell (*V*_*V*_(*lb/typeII*)) was estimated by point counting. For each parameter, at least 130 counting events were generated per lung to ensure that the total observed experimental variability was dominated by the biological variability among the individuals under study and not by the variability among stereological measurements within one individual [20].

### Statistics

Data are expressed as mean ± SD. Data were analyzed by the double-sided parametric t-test for independent samples. A p value < 0.05 was considered significant.

## Results

### Functional data of lavaged animals

**Figure 1:**
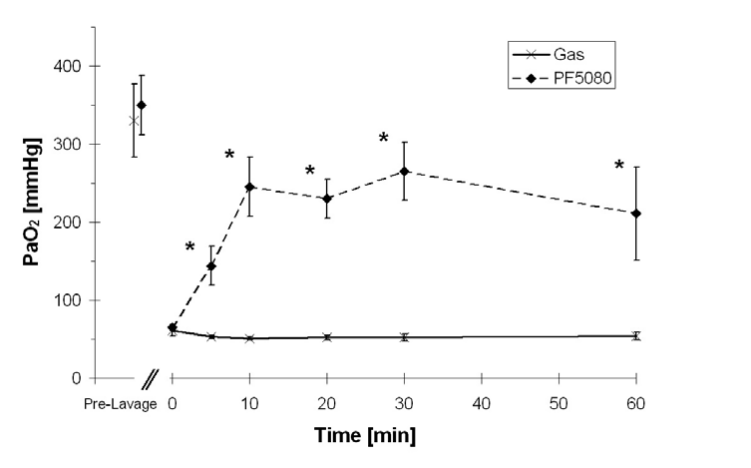
**Arterial tension of oxygen in lavaged animals**. Mean ± SD of arterial tension of oxygen (PaO_2_) in gas (X) and liquid (◆) ventilated animals prior to lavage (pre-lavage), after lavage (base line, 0 min) and during the subsequent hour of experiment. Values in the PF5080 group are significantly higher than in gas ventilated animals (* p < 0.0001).

All animals survived the lavage procedure and the subsequent 1 hour of treatment without any significant disturbances. Thus, a complete set of data was obtained from all 10 animals. Hemodynamic parameters remained stable throughout the experimental period (data not shown). Surfactant depletion by BAL caused a significant drop in arterial oxygenation (Fig. 1) and an increase in PaCO_2 _despite of an increase in PIP and subsequently higher tidal volumes (Tab. 1). A significant improvement in oxygenation was found within 5 minutes after starting partial liquid ventilation. At 30 minutes, tidal volume was significantly higher in the PLV group when compared with conventionally ventilated animals. At the end of the study (60 minutes) no significant differences between groups were found for tidal volume and PaCO_2 _(Tab. 1).

**Table 1:** Ventilatory and blood gas parameters of ventilated animals

	**Tidal volume **[ml/kg]		**PaCO_2 _**[mmHg]	
*Time point*	Gas	PF5080	Gas	PF5080

*Pre-lavage*	16.3 ± 7.1	15.4 ± 3.2	39 ± 7	42 ± 4
*After lavage*	18.1 ± 5.2	18.9 ± 1.1	55 ± 6	56 ± 7
*30 min therapy*	15.4 ± 4.1	20.9 ± 0.9^†^	56 ± 9	44 ± 9
*60 min therapy*	18.5 ± 4.1	22.7 ± 2.6	48 ± 3	44 ± 9

Data are presented as mean ± SD. ^†^p < 0.05 PF5080 vs. Gas.

### Qualitative microscopical findings

**Figure 2:**
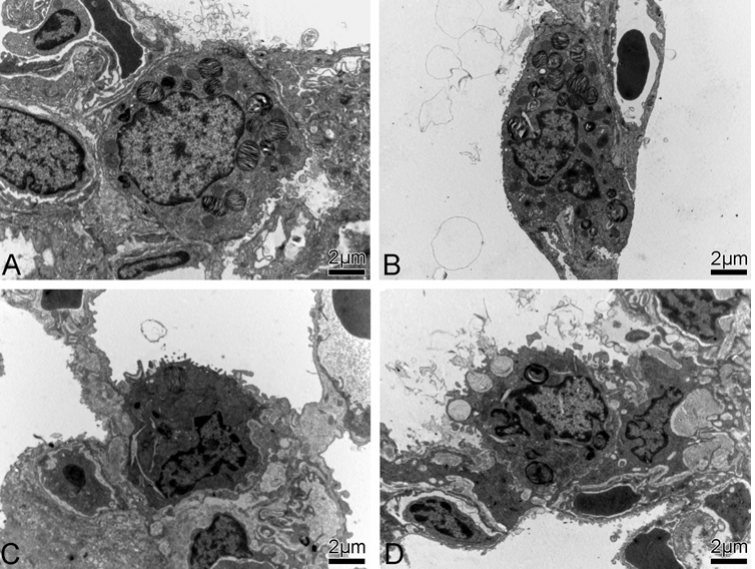
**Type II cell ultrastructure**. Transmission electron micrographs demonstrating type II cell ultrastructure in Healthy-Gas (A), Healthy-PF5080 (B), Lavaged-Gas (C), and Lavaged-PF5080 (D) groups. Qualitatively, the lamellar bodies appear normal in number in the Healthy-Gas (A) and Healthy-PF5080 (B) groups, while the type II cells seem to be smaller in size and contain less lamellar bodies in the Lavaged-Gas (C) and Lavaged-PF5080 (D) groups. Lamellar bodies in the process of secretion were seen more frequently in the lavaged animals (exemplarly shown in D).

**Figure 3:**
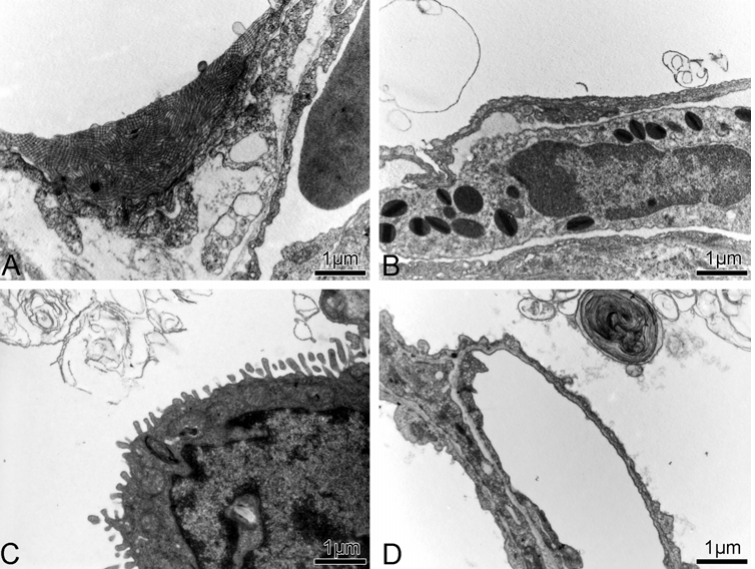
**Intraalveolar surfactant ultrastructure**. Transmission electron micrographs demonstrating intraalveolar surfactant ultrastructure in Healthy-Gas (A), Healthy-PF5080 (B), Lavaged-Gas (C), and Lavaged-PF5080 (D) groups. The presence of tubular myelin with its characteristic lattice-like structure in the healthy animals is exemplarly shown in (A). Multi- and unilamellar surfactant forms are shown in (B). Tubular myelin was only extremely rarely seen in the lavaged animals where multi- and unilamellar forms (C) and numerous lamellar body-like forms (D) were present.

The light microscopic appearance of the lungs showed no major differences between groups (not shown), thus requiring electron microscopic analysis. Representative electron micrographs demonstrate the ultrastructural appearance of type II cells and intracellular surfactant-storing lamellar bodies (Fig. 2) and intraalveolar surfactant (Fig. 3) in the four groups. The lamellar bodies within type II cells were filled with tightly packed intracellular surfactant material. All intraalveolar surfactant subtypes could be found.

No major differences between gas and PF5080 filled lungs with regard to type II cells and intraalveolar surfactant subtypes in the healthy (Fig. 2A, 2B, 3A, 3B) as well as in the lavaged groups (Fig. 2C, 2D, 3C, 3D) could be seen. However, when compared to healthy lungs, the lavaged groups had more type II cells with lamellar bodies in the process of secretion (Fig. 2C, 2D). Within the alveolar lumen, freshly secreted lamellar body-like surfactant forms were most prominent in both lavaged groups (Fig. 3C, 3D) while tubular myelin was virtually absent.

### Stereological data

**Figure 4:**
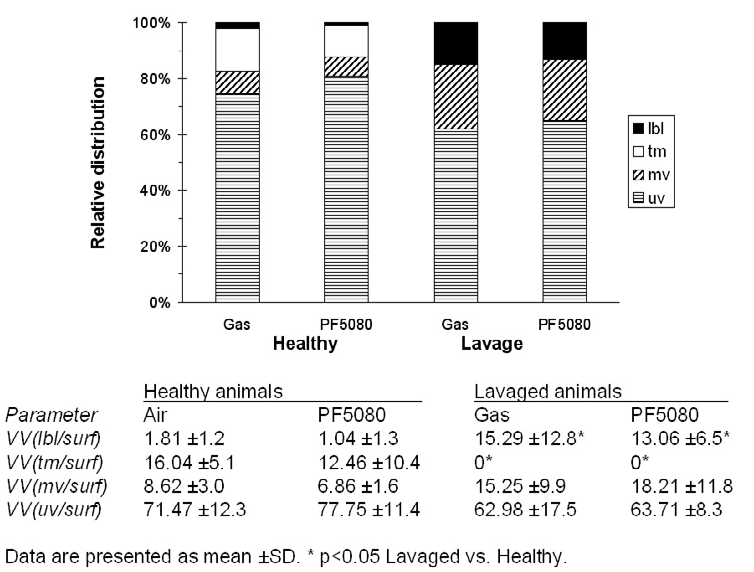
**Composition of intraalveolar surfactant**. Relative composition of intraalveolar surfactant in the four groups. Clear differences were noted between healthy and lavaged animals, irrespective whether they were filled with gas or PF5080. While all four different intraalveolar surfactant subtypes (lamellar body-like forms = lbl, tubular myelin = tm, multilamellar vesicles = mv, unilamellar vesicles = uv) were present in healthy animals, there were no measurable amounts of tubular myelin and decreased fractions of unilamellar vesicles in lavaged animals. This was counterbalanced by increased fractions of lamellar body-like forms and multilamellar vesicles in the lavaged groups, indicating a relative increase in freshly secreted surfactant material in the alveoli.

The stereological data are summarized in Tables 2, 3, 4. Data characterizing parenchymal architecture (Tab. 2), type II cells and lamellar bodies (Tab. 3) and intraalveolar surfactant content (Tab. 4) and its composition (Fig. 4) are given.

**Table 2:** Stereological data on parenchymal architecture

	**Healthy animals**		**Lavaged animals**	
*Parameter*	Gas	PF5080	Gas	PF5080

*V*(*lung*) [cm^3^]	5.00 ± 0.8	4.42 ± 1.7	3.76 ± 0.3*	5.34 ± 1.1^†^
*V*_*V*_(*par/lung*)	0.94 ± 0.02	0.95 ± 0.03	0.92 ± 0.04	0.95 ± 0.02
*V*(*sep*) [cm^3^]	1.4 ± 0.2	1.1 ± 0.3	1.0 ± 0.2*	1.3 ± 0.2
τ¯ MathType@MTEF@5@5@+=feaafiart1ev1aaatCvAUfKttLearuWrP9MDH5MBPbIqV92AaeXatLxBI9gBaebbnrfifHhDYfgasaacH8akY=wiFfYdH8Gipec8Eeeu0xXdbba9frFj0=OqFfea0dXdd9vqai=hGuQ8kuc9pgc9s8qqaq=dirpe0xb9q8qiLsFr0=vr0=vr0dc8meaabaqaciaacaGaaeqabaqabeGadaaakeaaiiGacuWFepaDgaqeaaaa@2E90@(*sep*) [μm]	5.7 ± 1.3	6.0 ± 1.0	4.1 ± 1.0	4.1 ± 0.4*
*S*(*alvepi*) [m^2^]	0.25 ± 0.1	0.19 ± 0.1	0.24 ± 0.04	0.33 ± 0.1*
ν¯V MathType@MTEF@5@5@+=feaafiart1ev1aaatCvAUfKttLearuWrP9MDH5MBPbIqV92AaeXatLxBI9gBaebbnrfifHhDYfgasaacH8akY=wiFfYdH8Gipec8Eeeu0xXdbba9frFj0=OqFfea0dXdd9vqai=hGuQ8kuc9pgc9s8qqaq=dirpe0xb9q8qiLsFr0=vr0=vr0dc8meaabaqaciaacaGaaeqabaqabeGadaaakeaaiiGacuWF9oGBgaqeamaaBaaaleaacqWGwbGvaeqaaaaa@2FE4@(*air*) [10^3 ^μm]	98.7 ± 1.6	95.3 ± 3.7	88.8 ± 15.7	81.6 ± 20.6

*Abbreviations*: *V*(*lung*) = total lung volume; *V*_*V*_(*par/lung*) = volume fraction of parenchyma; *V*(*sep*) = total volume of alveolar septal tissue; τ¯
 MathType@MTEF@5@5@+=feaafiart1ev1aaatCvAUfKttLearuWrP9MDH5MBPbIqV92AaeXatLxBI9gBaebbnrfifHhDYfgasaacH8akY=wiFfYdH8Gipec8Eeeu0xXdbba9frFj0=OqFfea0dXdd9vqai=hGuQ8kuc9pgc9s8qqaq=dirpe0xb9q8qiLsFr0=vr0=vr0dc8meaabaqaciaacaGaaeqabaqabeGadaaakeaaiiGacuWFepaDgaqeaaaa@2E90@(*sep*) = mean alveolar septal thickness; *S*(*alvepi*) = alveolar epithelial surface area; ν¯V
 MathType@MTEF@5@5@+=feaafiart1ev1aaatCvAUfKttLearuWrP9MDH5MBPbIqV92AaeXatLxBI9gBaebbnrfifHhDYfgasaacH8akY=wiFfYdH8Gipec8Eeeu0xXdbba9frFj0=OqFfea0dXdd9vqai=hGuQ8kuc9pgc9s8qqaq=dirpe0xb9q8qiLsFr0=vr0=vr0dc8meaabaqaciaacaGaaeqabaqabeGadaaakeaaiiGacuWF9oGBgaqeamaaBaaaleaacqWGwbGvaeqaaaaa@2FE4@(*air*) = volume-weighted mean volume of distal air spaces.

Data are presented as mean ± SD. * p < 0.05 Lavaged vs. Healthy. ^† ^p < 0.05 PF5080 vs. Gas.

**Table 3:** Stereological data on alveolar type II cells and intracellular surfactant

	**Healthy animals**		**Lavaged animals**	
Parameter	Gas	PF5080	Gas	PF5080

*N*(*typeII/lung*) [10^6^]	229.3 ± 85.1	220.3 ± 72.5	209.1 ± 49.8	285.7 ± 62.6
ν¯N MathType@MTEF@5@5@+=feaafiart1ev1aaatCvAUfKttLearuWrP9MDH5MBPbIqV92AaeXatLxBI9gBaebbnrfifHhDYfgasaacH8akY=wiFfYdH8Gipec8Eeeu0xXdbba9frFj0=OqFfea0dXdd9vqai=hGuQ8kuc9pgc9s8qqaq=dirpe0xb9q8qiLsFr0=vr0=vr0dc8meaabaqaciaacaGaaeqabaqabeGadaaakeaaiiGacuWF9oGBgaqeamaaBaaaleaacqWGobGtaeqaaaaa@2FD4@(*typeII*) [μm^3^]	385.7 ± 27.2	391.5 ± 27.5	344.7 ± 43.9	337.8 ± 16.3*
*V*(*lb/typeII*) [μm^3^]	61.3 ± 14.1	49.9 ± 5.5	26.9 ± 7.1*	27.9 ± 1.7*
*V*(*lb/lung*) [mm^3^]	14.0 ± 6.3	11.0 ± 4.0	5.5 ± 1.5*	8.0 ± 2.0
*V*_*V*_(*lb/mm^3 ^par*) [10^6 ^μm^3^]	2.9 ± 0.9	2.7 ± 0.4	1.6 ± 0.3*	1.6 ± 0.3*

*Abbreviations*: *N*(*typeII/lung*) = number of type II cells per lung; ν¯N
 MathType@MTEF@5@5@+=feaafiart1ev1aaatCvAUfKttLearuWrP9MDH5MBPbIqV92AaeXatLxBI9gBaebbnrfifHhDYfgasaacH8akY=wiFfYdH8Gipec8Eeeu0xXdbba9frFj0=OqFfea0dXdd9vqai=hGuQ8kuc9pgc9s8qqaq=dirpe0xb9q8qiLsFr0=vr0=vr0dc8meaabaqaciaacaGaaeqabaqabeGadaaakeaaiiGacuWF9oGBgaqeamaaBaaaleaacqWGobGtaeqaaaaa@2FD4@(*typeII*) = number-weighted mean volume of type II cells; *V*(*lb/typeII*) = total volume of lamellar bodies per type II cell; *V*(*lb/lung*) = total volume of lamellar bodies per lung; *V*_*V*_(*lb/mm^3 ^par*) = volume density of lamellar bodies per mm^3 ^parenchyma. Data are presented as mean ± SD. * p < 0.05 Lavaged vs. Healthy.

**Table 4:** Stereological data on intraalveolar surfactant

	**Healthy animals**		**Lavaged animals**	
Parameter	Gas	PF5080	Gas	PF5080

*S*_*S*_(*surf/alvepi*) [%]	21.9 ± 4.1	19.9 ± 4.8	8.0 ± 1.9*	8.6 ± 2.2*
*V*(*surf/lung*) [mm^3^]	21.1 ± 10.0	19.3 ± 3.6	10.0 ± 2.4*	13.3 ± 5.2
*V*_*V*_(*surf/mm^3 ^par*) [10^6 ^μm^3^]	4.4 ± 1.6	5.0 ± 1.6	2.9 ± 0.5	2.6 ± 0.7*

*Abbreviations*: *S*_*S*_(*surf/alvepi*) = surface fraction of alveolar epithelium covered with surfactant; *V*(*surf/lung*) = total volume of intraalveolar surfactant per lung; *V*_*V*_(*surf/mm^3 ^par*) = volume density of intraalveolar surfactant per mm^3 ^parenchyma.

Data are presented as mean ± SD. * p < 0.05 Lavaged vs. Healthy.

Comparison of PF5080 and gas filled lungs revealed a significantly higher total lung volume in lavaged, PF5080 filled animals (Tab. 2). In all other aspects, there were no quantitative differences between gas and PF5080 filled lungs neither in the healthy nor lavage group.

However, there were clear differences between healthy and lavaged lungs, irrespective whether they were filled with gas or PF5080. The mean values for the volume-weighted mean volume of distal airspaces were not different between groups (Tab. 2). Surfactant depletion by BAL caused a decrease in type II cell volume due to a decrease in the volume of lamellar bodies per cell with a subsequent decrease in the intracellular surfactant content per lung (Tab. 3). Taking the qualitative findings and the stereological data on intraalveolar surfactant into account, this was most probably due to an increased secretion of lamellar bodies in these groups.

The fraction of the alveolar epithelial surface area that was covered with surfactant as well as the total intraalveolar surfactant content per lung was significantly smaller in lavaged animals (Tab. 4). Lavage did not only affect intraalveolar surfactant content but also its composition (Fig. 4). In contrast to the considerable amount of tubular myelin in healthy lungs, tubular myelin was very rarely found in lavaged animals. The amount of tubular myelin was too low to generate counting events during stereological analysis in these groups. This lack of tubular myelin was counterbalanced by a higher volume fraction of lamellar body-like forms and multilamellar vesicles in the lavaged animals, indicating a relative increase in the fraction of freshly secreted surfactant in the alveoli.

## Discussion

Pulmonary surfactant prevents end-expiratory alveolar collapse. Bronchoalveolar lavage induces surfactant deficiency and has been intensively used in animal models to study pharamcological agents or ventilatory strategies [2]. Whereas changes that are induced by BAL and mechanical ventilation in physiological parameters are well studied, little was known concerning the quantitative changes in the intraalveolar and intracellular surfactant composition.

Intraalveolar perfluorocarbons prevent end-expiratory alveolar collapse and thus, improve the BAL induced respiratory insufficiency. PFC seem to increase surfactant secretion [7,8], however, it was not known how very short term liquid ventilation with PF5080 alters surfactant composition in a BAL induced animal model.

The present study in ventilated animals, for the first time, quantifies effects of BAL and subsequent PF5080 administration upon intracellular and intraalveolar surfactant, using transmission electron microscopy and stereology.

### Surfactant changes caused by bronchoalveolar lavage

The BAL procedure resulted – as intended – in a marked decrease in intraalveolar surfactant content associated with changes in its relative composition. Within intraalveolar subtypes, there was a relative decrease in tubular myelin and a relative increase in lamellar body-like forms which was most probably due to an increased secretion of lamellar bodies into the alveoli stimulated by the lack of surface active surfactant forms. Since SP-A is necessary for the transformation of lamellar body-like forms into tubular myelin [22], it is possible that a lack of functionally active SP-A is involved in these changes in intraalveolar surfactant composition.

The BAL model is commonly used to simulate the clinical situation of surfactant deficiency and to test the efficacy of different therapeutic strategies [2]. Whereas it is well known that BAL causes a surfactant depletion the quantitative effects upon intracellular surfactant composition and content were not known up until now. As the results show, the amount of intracellular lamellar bodies – the storage organelle of surfactant – is only about half as high as in healthy animals.

### Surfactant synthesis and secretion during liquid ventilation

Data upon the impact of perfluorocarbons on the pulmonary surfactant system are controversial and seem to depend on the type of PFC that is studied. In spontaneously breathing rats submersed in FC-75 for 3 hours the pulmonary surfactant content did not differ from air breathing control animals [9]. During the transition period from liquid to air breathing pulmonary compliance deteriorated, more likely due to an interaction of PFC with the surface tension lowering properties than with surfactant metabolism [23]. In preterm minipigs liquid ventilation did not alter the incorporation of acetate and choline into the lung and synthesis of lecithin seemed not different between conventional and liquid ventilation [24]. In preterm rabbits [25] and preterm lambs [26] the content of saturated phosphatidylcholine in lung lavage material did not change after one hour of liquid ventilation. Using labeled choline, Steinhorn and colleagues showed a higher content of labeling in the lung and BAL of perfluobron treated animals when compared with conventional gas ventilation [8]. However, interpretation of the data is difficult for several reasons [27]. The intraalveolar presence of PFC will prevent a complete removal of surfactant by lavage [28], making BAL material less reliable to estimate intraalveolar surfactant content in liquid ventilated animals. Furthermore BAL does not allow conclusions concerning intracellular surfactant synthesis. An increased surfactant content could be due to an increased synthesis or reduced degradation of intraalveolar surfactant [27].

To clarify PFC-surfactant interaction we recently studied the effect of different PFC upon surfactant synthesis and secretion in isolated type II pneumocytes and found a PFC mediated increase in surfactant secretion, but a decreased phospholipid synthesis [7]. Whereas the increased secretion would be in accordance with data found by Steinhorn et al. in healthy animals [8], the *in vivo *impact of PFC upon intracellular surfactant synthesis remained speculative.

The present study of short term liquid ventilation in surfactant depleted animals shows that intracellular and intraalveolar surfactant content and composition was not different between liquid and gas ventilated animals. The data suggest that very short term PLV with PF5080 did neither increase surfactant secretion nor decrease surfactant synthesis when compared with conventional mechanical ventilation. Whereas Steinhorn et al. used healthy animals [8], PLV was performed in lavaged animals in the present study. BAL causes an intraalveolar surfactant depletion and thus induces a very strong stimulus for surfactant secretion. Therefore, effects of PFC induced secretion could be less prominent in an animal model of BAL.

Interestingly, the present *in vivo *study did not show any alterations in intracellular surfactant pool size. Several points have to be considered to explain the difference to previous *in vitro *data [7]. Firstly, the current experimental setting describes the cumulative effects of PLV upon surfactant metabolism. Secondly, under *in vitro *conditions PFC come into direct contact with isolated type II pneumocytes and can therefore alter cellular metabolism [29]. The direct PFC cellular contact is prevented *in vivo *by forming PFC emulsions [30,31] that suppress direct PFC effects [15]. Thirdly, the lavage induced surfactant depletion represents a strong stimulus for surfactant synthesis and can therefore "override" the suggested PFC induced inhibition of surfactant synthesis. Finally, variations in lipid solubility of different PFC affect cellular activity [29]. Thus, the *in vivo *impact upon pulmonary surfactant metabolism is likely to vary with different PFC, as it has been shown *in vitro *[7]. To further investigate the complex interaction between PFC and surfactant metabolism additional studies in healthy animals using different PFC types are required.

### Electron microscopical and stereological surfactant analysis

In experimental studies, surfactant analysis is usually performed on material obtained by bronchoalveolar lavage. However, only intraalveolar surfactant can be harvested by this approach. In comparison, a morphological approach by transmission electron microscopy and stereology, as performed in the present study, allows a qualitative and quantitative analysis of the intraalveolar as well as the intracellular surfactant compartment preserved in its natural microorganization and localization within the lung [10,13]. To preserve the alveolar lining layer, chemical fixation "from behind", i.e. vascular perfusion fixation, instead of instillation fixation via the airways should be performed [32,33]. However, even under carefully controlled experimental conditions, only about 20% of the alveolar surface are found to be covered with surfactant after perfusion fixation [10]. Although physical fixation by freezing demonstrates a continuous alveolar lining layer [34], it preserves only very thin tissue layers of 20–200 μm thickness, making this approach unsuitable for stereological studies where a sampling protocol is required that generates samples that are representative for the whole organ. Stereological studies therefore rely upon homogenous and reproducible fixation of the whole lung which, at present, can only be achieved by chemical fixation [35]. An alternative approach to vascular perfusion is based on a non-aqueous fixation by osmium tetroxide dissolved in perfluorocarbon. This method, introduced by Sims and coworkers for the mucus lining of the trachea [36], has been refined to study the surfactant film of the alveoli and airways [37].

### Morphological correlate of BAL and partial liquid ventilation effects

Several studies investigated the impact of BAL [2] and liquid ventilation upon pulmonary histology. However, only the present study used a design-based stereological approach to formally quantify histological changes in surfactant depleted rats. Recently van Eeden et al. [38] investigated the effects of PLV after surfactant depletion in a rabbit model by morphometry at the light microscopic level and by qualitative electron microscopy. By reporting the number of type II cell profiles per field of vision, the authors concluded that conventional mechanical ventilation results in a lower number of type II cells when compared with partial liquid ventilation. However, besides differences in the experimental conditions, this seeming difference to our results is most probably due to differences in the methods used for quantification. The number of cell profiles per field of vision is not directly related to the number of cells in an organ [20]. Due to higher chances for bigger cells of being hit in a thin histological section, cell profiles per field of vision do not represent an unbiased sample. Instead, only design-based stereology, by using the disector method as a counting probe, allows to report unbiased data on the total number of cells within the lung [20,21].

## Conclusion

The present study quantifies effects of a commonly used experimental procedure – surfactant depletion by bronchoalveolar lavage – on the intra- and extracellular surfactant content and subtype composition. According to the present data the amount of intracellularly stored surfactant is about half as high after BAL as in healthy animals. In lavaged animals intratracheal application of PF5080 and subsequent very short term liquid ventilation did not alter intra- or extracellular surfactant content or subtype composition.

## Competing interests

The author(s) declare that they have no competing interests.

## Authors' contributions

MR has made substantial contribution to the conception and design of the study, performed the animal experiments and wrote the first draft of the manuscript.

SW performed the histological analysis, calculated the data and made substantial contribution to the analysis and interpretation of the data.

LK performed the histological analysis, calculated the data and made substantial contribution to the analysis and interpretation of the data.

WB contributed to the conception and design of the study, performed the animal experiments and revised the manuscript carefully.

RRW contributed to the conception of the study and the data interpretation, and revised the manuscript critically.

MO has made substantial contribution to the conception and design of the study; organized, performed and supervised histological analysis, made substantial contribution to data analysis and interpretation and to the final manuscript.
